# Maternal body mass index change as a new optimal gestational weight gain predictor in overweight women

**DOI:** 10.3325/cmj.2019.60.508

**Published:** 2019-12

**Authors:** Zoran Meštrović, Damir Roje, Ajka Relja, Indira Kosović, Nađa Aračić, Marko Vulić, Ozren Polašek

**Affiliations:** 1 Department of Obstetrics and Gynecology, Clinical Hospital Center Split, Split, Croatia; 2 Department of Public Health, University of Split School of Medicine, Split, Croatia; 3 Polyclinic Cito, Split, Croatia

## Abstract

**Aim:**

To investigate the relationship between maternal pre-pregnancy body-mass index (BMI) and neonatal birth weight.

**Methods:**

The observational study included 2906 mothers and their neonates born from 2005 to 2011 at the Department of Gynecology and Obstetrics, Split University Hospital Center. Mothers with singleton term pregnancies who were overweight before pregnancy (BMI 25-29.9 kg/m^2^) were compared with those with normal pre-pregnancy weight (BMI 18.5-24.9 kg/m^2^). BMI change was assessed as a predictor of birth weight, categorized as small (SGA), appropriate (AGA), or large for gestational age (LGA).

**Results:**

The rate of SGA infants was significantly lower (n = 199; 6.8% vs n = 1548; 9.2%) and the rate of LGA infants significantly greater among pre-pregnancy overweight mothers compared with normal-weight mothers (n = 371; 12.8% vs n = 1302; 7.8%; *P* < 0.001 both). Overweight mothers had a significant probability of delivering an SGA neonate when they gained less than 6 kg, as compared with 8 kg among normal-weight mothers. They had a significant probability of delivering an LGA neonate when they gained more than 14 kg, compared with more than 20 kg among normal-weight mothers. BMI change was a more consistent indicator, suggesting that the ranges of 3.0-7.9 kg/m^2^ in normal-weight and 2-5.9 kg/m^2^ in overweight women were not associated with a significant increase in the rate of SGA or LGA.

**Conclusion:**

Maternal height seems to be an important factor in optimal weight gain definition, suggesting that BMI change should be a preferred measure of pregnancy-related weight.

Overweight and obesity are major risk factors for morbidity and mortality, reflecting their considerable public health importance. Over the last few decades, their prevalence has surged irrespective of age, sex, ethnicity, or education level (1). Obesity has now reached truly epidemic proportions, affecting from one-third to more than half of reproductive age women in developed countries (2,3).

Optimal weight gain estimate for women during pregnancy still presents a substantial enigma. It has been shown that greater gestational weight gain decreases some perinatal risks (eg, preterm delivery or fetal growth retardation), while increasing some others (eg, preeclampsia or macrosomia) (2-7). The Institute of Medicine (IOM) in 2009 developed recommendations aligning the desired weight gain to the World Health Organization (WHO) body mass index (BMI) categories (8). Although this approach seemed intuitive, it has often been criticized (8). The simple mathematical weight gain formula is just an approximation of the biological and developmental processes, but still remains the most widely used formula so far.

In the present study, we calculated weight gain for pre-pregnancy overweight women as expressed as maternal BMI change, thus relying more on height than on weight gain compared with previous recommendations. In the beginning, recommendations were regularly applicable to all pregnant women without distinction, irrespective of their anthropometric characteristics. In 1990, the IOM proposed the calculation on the basis of the woman's pre-pregnancy BMI as a classification input criterion (9). This concept was improved and in 2009, when the same institution issued a revised protocol following BMI categorization according to the WHO classification (8). The objective of the new guidelines was to reduce the unfavorable health effects of inadequate or excessive gestational weight gain on the mother, pregnancy, and infant. A positive and independent correlation of maternal body height and pre-pregnancy BMI with gestational weight gain has long been demonstrated (10). However, BMI can easily have the same value in extremely tall and extremely short women if they have symmetrically inverse body weights. Therefore, the use of BMI alone may introduce a bias, as it may not reflect the body size and predicted changes over the course of pregnancy in the same way for different women. We offer weight gain recommendations for each category in BMI change range values that can be precisely recalculated into kilograms for any woman according to her height. We hypothesized that BMI change would better reflect pregnancy-related weight gain, which reflects fetal growth assessment as the main perinatal outcome measure.

## Methods

### Participants

The study included 2906 overweight mothers and their neonates born during a 7-year period (2005-2011) at the Department of Gynecology and Obstetrics, Split University Hospital Center in Split, Croatia. Almost all citizens of the Split-Dalmatia County are of European origin and are predominantly Croats (96.3%).

### Inclusion and exclusion criteria

We only included pre-pregnancy overweight mothers according to the WHO standards (BMI 25-29.9 kg/m^2^) with singleton term pregnancies (37th to 42nd week of gestation). Pregnancies with normal pre-pregnancy weight (BMI 18.5-24.9 kg/m^2^) were used as a comparison group. Exclusion criteria were pregnancies complicated with diabetes mellitus type 1 or 2, gestational diabetes mellitus, Rh or other immunization, fetal hydrops, neonatal malformations, stillbirths or early neonatal deaths, and incomplete medical documentation. The pregnancies were selected from a larger cohort study that included 21 400 newborns during the same follow-up period (2005-2011) (7).

### Maternal data

Data on maternal height, pre-gestational weight, and weight on the day of delivery were obtained from medical history. Body mass was expressed in kilograms, rounded to the nearest kilogram value. Body height was expressed in centimeters, rounded to the nearest centimeter value. Gestational age was expressed in completed weeks and calculated according to the first day of the last menstruation, corrected by ultrasonic assessment when discrepancy exceeded one week. Maternal BMI was calculated as follows: BMI (kg/m^2^) = body mass (kg)/body height^2^ (m^2^).

### Neonatal data

All neonates were weighed immediately upon birth on the same scale (Libela, Celje, Slovenia). Body mass below the 10th percentile was considered as small for gestational age (SGA), above the 90th percentile as large for gestational age (LGA), and between these two values as appropriate for gestational age (AGA). Neonatal ponderal index (PI) was calculated by the formula: PI (g/cm^3^) = birth weight (g) × 100/birth length^3^ (cm^3^). PI below the 10th percentile was considered as low (“neonatal thinness”) and above the 90th percentile as high (“neonatal obesity”), representing asymmetrical growth. The fetal growth charts were developed in the same institution, thus ensuring evaluation quality (11,12).

### Weight gain categorization

Maternal weight gain was expressed in two ways: in kilograms (2 kg increment) and as BMI change (1 kg/m^2^ increment). The relationship between the measured values and the presumed prevalence of LGA and SGA neonates (10% for a particular category) was calculated. The study was approved by the Ethics Committee of the Clinical Hospital Center Split.

### Statistical analysis

The χ^2^-test was used to analyze the dependence of the qualitatively categorized variables. Statistical data analysis was performed by the Statistica 7.0 software (StatSoft, Tulsa, OK, USA). The level of statistical significance was set at *P* < 0.05.

## Results

The study involved 2906 overweight women, accounting for 13.6% of the investigated population (N = 21 400). The proportion of underweight women (BMI<18.5 kg/m^2^) was 5.6% (n = 1205), of normal-weight women 78.3% (n = 16 751), and of obese women (BMI>30 kg/m^2^) 2.5% (n = 542).

Exclusion criteria were recorded in 67 mother-neonate pairs in the overweight group, as follows: incomplete medical documentation (n = 43), fetal hydrops (n = 3), malformations (n = 14), stillbirths (n = 5), and early neonatal death (n = 2).

Seventy-five (2.58%) women in the investigated group reported regular cigarette smoking. Only 24 (0.82%) overweight women reported that they smoked ten or more cigarettes daily. Smoking mothers gave birth to 6 (8%) SGA, 62 (82.7%) AGA, and 7 (9.3%) LGA newborns.

Pre-pregnancy overweight women were by 1.6 years older (*t* = 15.5), by 14.3 kg heavier (*t* = 67.9), and by 1.2 cm shorter (*t* = 6.4; *P* < 0.001 all). The mean pre-pregnancy BMI difference decreased by the end of pregnancy (5.2 kg/m^2^ vs 4.7 kg/m^2^) but remained significant (*t* = 68.9; *P* < 0.001). The neonates born to overweight mothers were on average by 68 g heavier (*t* = 6.5), by 0.5 cm shorter (*t* = 4.5), and had higher PI (*t* = 4.0; *P* < 0.001 all). The main characteristics of investigated populations are presented in Table 1.

**Table 1:** **The** main characteristics of mother-newborn pairs

	Pre-pregnancy normal weight (N = 16 751) (BMI 18.5-24.9 kg/m^2^)	Pre-pregnancy overweight (N = 2906) (BMI 25-29.9 kg/m^2^)	*P*
Maternal age (years)	28.6 ± 5.1	30.2 ± 5.4	<0.001^‡^
Primigravidae	7982 (47.6)	861 (29.6)	<0.001^§^
Maternal pre-pregnancy weight (kg)	62.3 ± 5.6	76.6 ± 6.3	<0.001^‡^
Maternal body height (cm)	170.2 ± 5.4	169.0 ± 5.7	<0.001^‡^
Maternal pre-pregnancy body mass index (kg/m^2^)	21.5 ± 1.6	26.7 ± 1.9	<0.001^‡^
Maternal weight gain (kg)	14.9 ± 4.4	15.4 ± 5.1	0.920^‡^
Maternal body mass index at delivery (kg/m^2^)	27.1 ± 2.3	31.8 ± 2.5	<0.001^‡^
Gestation age at delivery (completed weeks)	39.8 ± 1.2	39.7 ± 1.6	0.117^‡^
Birth weight (g)	3580 ± 489.6	3648 ± 501.9	<0.001^‡^
Birth length (cm)	50.7 ± 2.0	51.2 ± 2.2	<0.001^‡^
Ponderal index at birth (g/cm^3^)	2.65 ± 0.2	2.74 ± 0.2	<0.001^‡^
Low ponderal index (<10th percentile) among small for gestational age newborns	393 (25.4)	42 (21.1)	0.219^§^
High ponderal index (<10th percentile) among large for gestational age newborns	127 (9.7)	35 (9.4)	0.865^§^

*BMI – body mass index.

†Data are presented as mean ± standard deviation or count (percent).

‡t-test for independent samples.

§χ^2^ test.

### Fetal growth evaluation according to birth weight standard

The rates of SGA and LGA newborns across the weight gain groups reflected an interesting pattern in overweight mothers. The rate of SGA neonates was increased in mothers with very small weight gains (up to 6 kg) (Table 2). Similarly, a non-significantly higher rate of LGA neonates was also recorded in mothers with smaller gains (4-6 kg), and a significantly higher rate in mothers with greater weight gains (≥14 kg) (Table 2). The use of BMI change indicated a similar pattern, with lower rates of SGA in the lowest weight gain group (<2 kg/m^2^) (Table 3). However, for LGA neonates, BMI change yielded a much more even pattern, with somewhat higher rates for medium gains (4-6 kg/m^2^) and significantly higher rates for gains greater than 6 kg/m^2^ (Table 3).

**Table 2:** Fetal growth evaluation of pre-pregnancy overweight and normal weight mothers in association to presumed frequencies (SGA 10%, AGA 80%, and LGA 10%) according to gestational weight gain in kilograms (kg)*^†^

	Pre-pregnancy normal weight (BMI 18.5-24.9 kg/m^2^)						Pre-pregnancy overweight (BMI 25-29.9 kg/m^2^)				
Δkg		SGA n (%)	AGA n (%)	LGA n (%)	total n (%)	*P*	SGA n (%)	AGA n (%)	LGA n (%)	total n (%)	*P*
0-3.9		6 (20.7)^†^	21 (72.4)	2 (6.9)	29 (100)	0.524	4 (26.7)^†^	8 (53.3)	3 (20.0)	15 (100)	0.511
4-5.9		31 (22.5)^†^	105 (76.1)	2 (1.4)	138 (100)	0.001	11 (17.2)^†^	43 (67.2)	10 (15.6)	64 (100)	0.190
6-7.9		86 (18.9)^†^	358 (78.7)	11 (2.4)	455 (100)	<0.001	15 (9.5)	131 (82.9)	12 (7.6)	158 (100)	0.704
8-9.9		169 (11.5)	1244 (84.4)	60 (4.1)	1473 (100)	<0.001	35 (9.7)	292 (80.9)	34 (9.4)	361 (100)	0.957
10-11.9		276 (10.7)	2165 (83.7)	145 (5.6)	2586 (100)	<0.001	29 (6.4)	386 (85.6)	36 (8.0)	451 (100)	0.070
12-13.9		295 (10.4)	2371 (83.8)	162 (5.8)	2828 (100)	<0.001	23 (4.8)	401 (83.9)	54 (11.3)	478 (100)	0.008
14-15.9		273 (7.9)	2933 (85.0)	241 (7.1)	3447 (100)	<0.001	33 (6.7)	389 (78.9)	71 (14.4)^†^	493 (100)	0.027
16-17.9		187 (8.5)	1804 (82.5)	197 (9.0)	2188 (100)	0.105	17 (5.5)	247 (80.2)	44 (14.3)^†^	308 (100)	0.042
18-19.9		119 (6.6)	1461 (81.6)	210 (11.8)	1790 (100)	0.001	17 (5.9)	227 (78.5)	45 (15.6)^†^	289 (100)	0.036
20-21.9		42 (5.7)	589 (80.3)	103 (14.0)^†^	734 (100)	0.001	5 (4.4)	87 (76.3)	22 (19.3)^†^	114 (100)	0.048
≥22		64 (5.9)	850 (78.5)	169 (15.6)^†^	1083 (100)	<0.001	10 (5.7)	125 (71.4)	40 (22.9)^†^	175 (100)	0.003
Total		1548 (9.2)	13901 (83.0)	1302 (7.8)	16751 (100)	<0.001	199 (6.8)	2336 (80.4)	371 (12.8)	2906 (100)	0.001

*BMI – body mass index; SGA – small for gestational age; AGA – appropriate for gestational age; LGA – large for gestational age.

†Pairwise comparison of the SGA or LGA with the AGA significant at the level of *P* < 0.05 when SGA or LGA proportion was greater than 10% (χ^2^ test).

**Table 3:** Fetal growth evaluation of pre-pregnancy overweight and normal weight mothers in association to presumed frequencies (SGA 10%, AGA 80%, and LGA 10%) according to gestational weight gain presented in BMI (kg/m^2^) enhancement

	Pre-pregnancy normal weight (BMI 18.5-24.9 kg/m^2^)					Pre-pregnancy overweight (BMI 25-29.9 kg/m^2^)				
ΔBMI	SGA n (%)	AGA n (%)	LGA n (%)	total n (%)	*P*	SGA n (%)	AGA n (%)	LGA n (%)	total n (%)	*P*
0-1.9	21 (23.1)^†^	67 (73.6)	3 (3.3)	91 (100)	0.028	7 (14.0)^†^	35 (70.0)	8 (16.0)	50 (100)	0.507
2-2.9	89 (16.2)^†^	445 (81.1)	15 (2.7)	549 (100)	<0.001	19 (9.8)	156 (80.9)	18 (9.3)	193 (100)	0.958
3-3.9	278 (10.8)	2131 (83.1)	154 (6.1)	2563 (100)	<0.001	46 (9.0)	420 (82.4)	44 (8.6)	510 (100)	0.622
4-4.9	414 (9.6)	3639 (84.4)	259 (6.0)	4312 (100)	<0.001	40 (5.5)	600 (82.7)	86 (11.8)	726 (100)	0.004
5-5.9	351 (8.1)	3627 (84.0)	339 (7.9)	4317 (100)	<0.001	39 (5.8)	546 (81.5)	85 (12.7)	670 (100)	0.008
6-6.9	244 (8.9)	2239 (81.8)	256 (9.3)	2739 (100)	0.239	22 (6.0)	292 (79.1)	55 (14.9)^†^	369 (100)	0.025
7-7.9	95 (7.3)	1068 (82.1)	138 (10.6)	1301 (100)	0.064	12 (5.4)	171 (77.4)	38 (17.2)^†^	221 (100)	0.026
≥8	56 (6.4)	685 (77.9)	138 (15.7)^†^	879 (100)	< 0.001	14 (8.4)	116 (69.5)	37 (22.1)^†^	167 (100)	0.025
Total	1548 (9.2)	13901 (83.1)	1302 (7.8)	16751 (100)	<0.001	199 (6.8)	2336 (80.4)	371 (12.8)	2906 (100)	0.000

*BMI – body mass index; SGA – small for gestational age; AGA – appropriate for gestational age; LGA – large for gestational age.

†Pairwise comparison of the SGA or LGA with the AGA significant at the level of *P* < 0.05 when SGA or LGA proportion was greater than 10% (χ^2^ test).

### Fetal growth evaluation according to ponderal index

In overweight women, the mean PI was higher (2.74 vs 2.65%; χ^2^ = 4.1; *P* < 0.001) and the rate of neonates with PI less than the 10th percentile was higher than expected (12.5%; χ^2^ = 8.03; *P* = 0.005). The rate of neonatal obesity (PI>90th percentile) was 8.8% (χ^2^ = 1.21; *P* = 0.27). In any subgroup, normal and overweight subgroups did not show significant differences in PI subcategories irrespective of weight gain measured in kilograms or BMI change (Table 1).

## Discussion

This study suggests that maternal BMI change could be a useful predictor of neonatal anthropometry and, consequently, an important factor for pregnancy-related outcome. This also means that the IOM standards could be revised to improve the approach used in daily clinical practice. When taken together, according to our study, optimal weight gain expressed as BMI change for pre-pregnancy overweight women ranges from 2 to 5.9 kg/m^2^. The range for normal weight women is a little higher and wider (3-7.9 kg/m^2^).

We are still not able to answer the simple question of optimal weight gain for each pregnant woman. Supported by the vast majority of authors, IOM chose fetal growth assessment as the representative perinatal outcome factor and developed overall guidelines based on it (8). Fetal growth directly or indirectly reflects most other perinatal outcome components, thus, the calculation of optimal gestational weight gains presents the best, or at least the least unfavorable, choice (8,9). This study followed the same outcome selection model, and using percentile fetal growth tables previously developed at the same institution, ensured quality evaluation and reduction of the potential bias (11).

Gestational weight gain exceeds the IOM recommended standards in the majority of pregnant women in industrialized countries. Even more importantly, in women who were overweight and obese before pregnancy (<52%) the likelihood of uncontrolled excessive gestational weight gain exceeds the standards 4-fold (13,14). This results in an increase in pregnancy complication rate associated with their basic obesity, (possibly) impaired metabolism, and/or excessive gestational weight gain (2,6,15). Despite individual BMI categories defined by the WHO standards and the expected population rate of 18%-25%, overweight women are rarely investigated separately from obese ones (1-3,10,16-18). Their proportions, body shape, and metabolism are likely different from obese women, but they are also not completely similar to women with normal BMI. In order to make a further conceptual step compared with the analysis of the merged groups, this study included only overweight women.

Many authors consider the IOM guideline as being too liberal, assuming that appropriate weight gain for overweight women during pregnancy should be 5-7 kg (3). The results of this study point to BMI change as a useful predictor that outperforms simple weight gain expressed in kilograms, supporting IOM suggestion for pre-pregnancy overweight women, and just extending the range by 1.0 kg in the lower and 1.5 kg in the upper limit. The introduction of BMI as an input variable by the IOM was a breakthrough. We do believe that the benefits thus achieved must not be denied and that BMI index should be used for the rest of pregnancy too. To our knowledge, as an output measure, BMI was used only once. Asplund et al (19), with a small sample of only 186 pregnant women, demonstrated that a BMI increase by more than 25% irrespective of its initial value increased the prevalence of macrosomia. In this study, we propose for the first time the idea of BMI change in the context of woman's height as the new output measure. For a woman of 160 cm, pre-pregnancy body mass range of 64-76.2 kg implies overweight, whereas for a woman of 180 cm the respective range is 81-96.8 kg. This means that according to the existing IOM guidelines, identical optimal gestational weight gain is recommended for a woman of 160 cm with pre-pregnancy body mass of 64 kg and a woman of 180 cm with pre-pregnancy body mass of 96.8 kg. In the former, the upper limit of recommended weight gain (11.5 kg) accounts for 18% of the initial body weight, whereas in the latter, the lower limit of recommended weight gain (7.0 kg) accounts for 7.2% of the initial body weight. Therefore, our proposal could be a welcome step forward in the individualization of the IOM recommendations, with special importance for very tall and short women.

The limitations of this study include rather small sample sizes in the group breakdown. Also, we only used newborn anthropometry as fetal growth evaluation. It is a limited outcome in terms of the overall clinical assessment, but previously published studies and IOM itself resolved the same dilemma in a similar way. We excluded some pregnancy-related pathological conditions, but intentionally only those that can drastically affect fetal anthropometry (fetal hydrops, malformed fetuses, and stillbirths). Women suffering from chronic diseases (autoimmune disorders, arterial hypertension) or other pregnancy complications, such as preeclampsia, were enrolled in the study as a constituent part of the overweight population.

One of the recently developed approaches to this topic includes a possible explanation for the inter-newborn variations. It has been suggested that the maternal nutritional status can have a causal effect on the epigenetic fetal profile. The gene coding for insulin growth factor 2 was found to be hypomethylated in the placentas from intrauterine growth restriction and/or SGA pregnancies (20). A significant association has also been reported between LGA infants and differential methylation of the glucocorticoid receptor gene in the placenta (21). Although these results are only the first step toward the understanding of the mechanics of the neonatal growth, they nevertheless show a highly promising line of evidence that might provide a more definitive answer to fetal growth patterns.

Based on the results of this study, we strongly believe that in optimal weight gain estimation/calculation, BMI can be used not only as an input but also as an output measure. The final recalculation of the recommended BMI change takes in account the woman's height. This proposal upgrades the IOM recommendations, with special significance in the tallest and shortest subgroups of women. It presents a step further in the efforts to find a formula for the calculation of the optimal gestational weight gain for each individual woman, thus providing conditions for the most favorable perinatal outcome.
